# Effects of Encapsulation and In Vitro Digestion on Anthocyanin Composition and Antioxidant Activity of Raspberry Juice Powder

**DOI:** 10.3390/foods14142492

**Published:** 2025-07-16

**Authors:** Mokgaetji Johanna Mokale, Sreejarani Kesavan Pillai, Dharini Sivakumar

**Affiliations:** 1 Phytochemical Food Network Research Group, Department of Crop Sciences, Tshwane University of Technology, Pretoria 0001, South Africa; mokgaetjimokale@gmail.com; 2 Centre for Nanostructures and Advanced Materials, DSI-CSIR Nanotechnology Innovation Centre, Council for Scientific and Industrial Research, Pretoria 0001, South Africa; skpillai@csir.co.za; 3 Centre for Nutrition & Food Sciences, Queensland Alliance for Agriculture and Food Innovation, The University of Queensland, Brisbane, QLD 4108, Australia

**Keywords:** berry fruits, cyanidin-3-O-glucoside, bio-accessibility, biopolymers

## Abstract

Microbeads of raspberry extract were produced using encapsulation matrices alginate + pea protein isolate + *psyllium mucilage*, alginate + pea protein isolate + *psyllium mucilage* + okra, and alginate + pea protein isolate + *psyllium mucilage* + *Aloe ferox* gel + gallic acid using freeze-drying method. The microbeads were characterised and assessed for their effectiveness on the release, bioaccessibility, of anthocyanin components and antioxidant activities during in vitro digestion. Alginate + pea protein isolate + *psyllium mucilage* + *Aloe ferox* gel + gallic acid matrix showed the highest encapsulation efficiency of 91.60% while the lowest encapsulation efficiency was observed in alginate + pea protein isolate + *psyllium mucilage* + okra (69.94%). Scanning electron microscope images revealed spherical shapes and varying surface morphologies for different encapsulation matrices. Despite the differences observed in Fourier transform infrared spectra, microbeads showed similar thermal degradation patterns. X-ray diffractograms showed amorphous structures for different encapsulation matrices. Comparatively, alginate+ pea protein isolate + *psyllium mucilage* + *Aloe ferox* gel + gallic acid microbeads exhibited the highest bioaccessibility for total phenols (93.14%), cyanidin-3-O-sophoroside (54.61%), and cyanidin-3-O-glucoside (55.30%). The encapsulation matrices of different biopolymer combinations (alginate+ pea protein isolate+ *psyllium mucilage*, alginate + pea protein isolate + *psyllium mucilage* + okra, and alginate + pea protein isolate + *psyllium mucilage* + *Aloe ferox* gel + gallic acid) enhanced anthocyanin stability and protected it against in vitro degradation of bioactive compounds.

## 1. Introduction

The raspberry, (*Rubus idaeus*), is well-known for its vivid colour and flavour, making it a popular commercial fruit in temperate regions. Raspberries are used in many culinary and health products for antioxidants, natural colourants, anthocyanins, and polyphenols [1]. Anthocyanins that make fruits red, and other polyphenols that protect against cardiovascular, metabolic, and neurodegenerative diseases [1]. Raspberry is used in a range of culinary and health products. The cyanidin-to-pelargonidin ratio of raspberries is 32:1 with 92.1 mg anthocyanins per 100 g [1].

However, anthocyanins are extremely sensitive to temperature, light, and pH, and can degrade during processing and storage [2]. Bio-accessibility is also crucial for anthocyanin health benefits [3]. A strong influence on anthocyanin stability and bioavailability can result from pH fluctuations and molecular modifications that can degrade in the digestive system resulting in reduced absorption [4]. Encapsulating anthocyanins improves their stability and bioaccessibility and is regarded as an effective strategy for preserving anthocyanins during digestion [5]. Techniques used for encapsulation include ionic gelation, spray drying, coacervation, freeze-drying, and emulsification [6]. Several polysaccharides and proteins have been used to encapsulate anthocyanins in berries [7].

Seke et al. [8] demonstrated the highest encapsulation efficiency (95.38%) using strawberry juice (SJ), pea protein, and *psyllium mucilage* (SJPPO). In the intestinal phase, cyanidin 3-glucoside, delphinidin, and malvidin 3-glucoside showed the highest bioaccessibility. The alginate-based encapsulation protects anthocyanins in acidic environments in the stomach [9]. However, the alginate matrix exhibits varying levels of porosity and permeability, influenced by the specific characteristics of the gelation conditions used [10]. Due to these variations, molecular diffusion rates can differ, limiting their effectiveness in food application. In the intestinal phase, alginate encapsulation might reduce anthocyanin bioavailability [11]. The combination of wall materials and encapsulation techniques is effective for both ionic gelation and complexation with cationic polyelectrolytes [12]. Polysaccharides and polymers such as sodium alginate, gallic acid, and pea protein isolate can interact electrostatically to initiate complexation [13]. The pH and ionic strength of the complexation process determine if these electrostatic compounds can be interchanged [14]. Proteins and hydrocolloids can enhance the safety of compounds encapsulated in gel particles by forming electrostatic interactions with anionic charges [13]. The low porosity of the gel matrix limits the passage of hydrophilic substances through its pores, thus providing protection.

Using hydrocolloids, protein isolates, and ionic gelation, anthocyanins have been encapsulated with promising results in terms of their stability, encapsulation efficiency, and bioaccessibility [15]. Despite ionic gelation being an effective and mild encapsulation method, the size and porosity of gel beads can impact release kinetics, potentially affecting bioavailability and reducing their effectiveness in targeted delivery applications [16]. Optimizing encapsulation techniques and overcoming associated challenges will be crucial to maximising raspberry anthocyanins’ potential in various products. One promising approach involves the combination of ionic gelation and complexation techniques, particularly with cationic polyelectrolytes [10]. The electrostatic interaction between sodium alginate, gallic acid, and pea protein isolate, for instance, has been shown to facilitate the formation of stable complexes [11]. However, while this approach has demonstrated potential, there remains a gap in our understanding of these encapsulation matrix configurations affect the chemical composition and biological functionality encapsulated sensitive actives, especially when subjected to varying pH and ionic strength conditions.

Our objective is to examine how raspberry polyphenolic extract encapsulated in various delivery matrices (alginate, pea protein isolate, *psyllium mucilage*, okra, *Aloe ferox* gel, and gallic acid) prepared by ionic gelation affects anthocyanin content, composition of cyanidin derivatives, and antioxidant activity. The study also assesses the bio-accessibility of raspberry bioactive compounds during digestion in vitro.

## 2. Materials and Methods

All standards, solvents, and chemicals were procured from Sigma Aldrich (Kempton Park, Johannesburg, South Africa). All chemicals used in this experiment were of analytical grade. Raspberry fruits were purchased from a commercial farm (Polokwane, South Africa).

### 2.1. Raspberry Anthocyanin Extraction and Alginate Beads Preparation

Raspberry fresh fruit was freeze-dried and ground into a fine powder. Raspberry anthocyanin extract was prepared according to Seke et al. [8] with slight modifications using 10 g of the raspberry powder andultrasonication (Digital ultrasonic bath, 40 kHz Ultrasonic Cleaner, Shanghai, China) using 80:20 ethanol/water (100 mL) for 30 min at 30 °C. The supernatant was centrifuged (Hermle Z326k, Hermle Labortechnik GmbH, Wehingen, German) at 5000× *g* for 20 min at 4 °C. The beads were formulated using a method described by Seke et al. [8]. Briefly, polysaccharide treatment combinations (Table 1) were dispersed in distilled water and agitated until completely homoginised. To ensure complete hardening of the microbeads, calcium chloride (5% *w*/*v*) was added to the raspberry extract. The polysaccharide solution was manually dropped into a raspberry extract solution (0.5% *w*/*v*) with a calcium chloride solution of 5% and left for 12 h to ensure complete hardening.

These microbeads were filtered using a Whatman #4 paper filter, allowing the filtrate to be recovered. The beads were then washed using distilled water, placed in −80 °C for 24 h, and freeze-dried (Benchtop Pro with Omnitronics, UK) for 7 days.

### 2.2. Total Anthocyanin Content (TAC) and Encapsulation Efficiency (EE%)

The total anthocyanin content (TAC) of beads was done following the method presented by Seke et al. [8] using, 1.5 mL of raspberry extract combined with 2.5 mL of 0.025 M potassium chloride buffer (pH 1) or 2.5 mL of 1 M sodium acetate buffer (pH 4.5). Results obtained of total anthocyanins were presented as mg equivalents of *cyanidin-3-glucoside* per g dry weight basis. TAC was calculated using Equations (1) and (2).

A = (A1 − A2) _pH 1.0_ − (A1 − A2) _pH 4.5_ (1)

(2) $$\mathrm{TAC}=\frac{A\;}{\varepsilon \;\times \;l\;}\times \;\;MW\times \;DF\times \;\;\frac{V}{W}\times \;\;1000$$

where TAC is the total anthocyanin content, A = absorbance, A1 = absorbance at 510 nm, A2 = absorbance at 700 nm, ε = extinction coefficient for cyanidin 3-glucoside (26,900), *MW* (molecular weight) = 449.2 g mol^−1^ for *cyanidin-3-glucoside*, *DF* = dilution factor; *V* = volume of the stock solution (mL), *W* = sample weight (mg); *l* = path length (1 cm), *M* extinction coefficient in L mol^−1^ cm^−1^ for cyanidin-3-glucoside, and 1000 is the conversion factor g to mg.

The encapsulation efficiency of anthocyanins was calculated using Equation (3).

(3) $$EE\;\left(\%\right)=\frac{Beads\;anthocyanin\;content}{Extract\;anthocyanin\;content}\times \;100$$

### 2.3. Scanning Electron Microscopy (SEM)

The microstructure of freeze-dried Raspberry powder and beads was studied using scanning electron microscopy (SEM) (Tescan Vega 3, Borno, Czech Republic) under the scanning electron microscope at magnifications of 2000, 2500, 5000, and 10,000×.

### 2.4. Fourier Transform Infrared Spectroscopy (FTIR) and X-Ray Diffraction

The chemical structure of anthocyanin-rich raspberry beads was analysed using an FT-IR spectrometer (Perkin Elmer Spectrum 100 spectrometer, Waltham, MA, USA). Following the methodology of Fathordoobady et al. [17] and Seke et al. [8] without any modification. The FT-IR spectra were recorded in the wavenumber range of 600–4000 cm^−1^. A total of 32 scans were performed with the spectra resolution maintained at 4 cm^−1^.

The crystallinity of beads was determined using X-ray diffraction (Panalytical X’pert PRO X-ray Diffractometer, Almelo, The Netherlands) according to the method described by Li et al. [18]. The microbead samples were placed in a sample holder, smoothed with a steel slide, and then positioned in the X-ray diffraction machine. The spectra were obtained using a 2θdegree angle from 10° to 90° with a step size of 0.0170 degrees using Cu-Ka radiation at a wavelength of 0.154 nm and the generator was set at 40 mA current with a 40 KV voltage.

### 2.5. In Vitro Digestion of Microbeads

**Figure 1:**
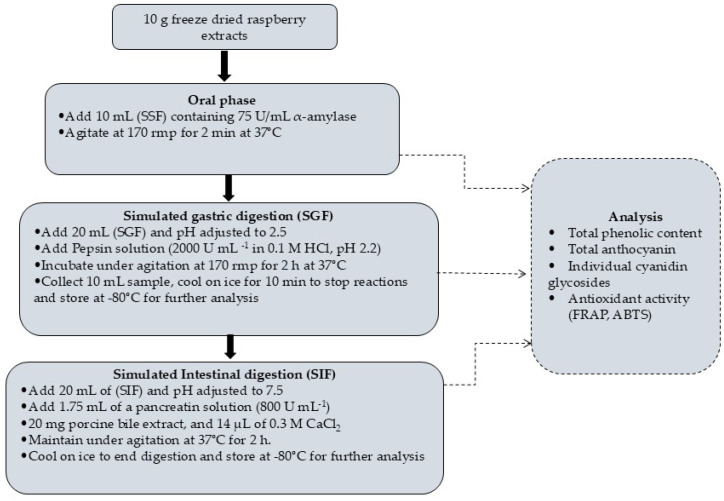
In vitro gastrointestinal digestion procedure of raspberry extracts.

The in vitro release properties of beads were determined using a method described by Seke et al. [8] with three stages. The three stages of digestion were done following a technique ascribed by Brodkorb et al. [19], such as undigested sample, gastric digested, and intestinal digested. Initially, in the simulated salivary fluid (SSF) phase, 10 mL of salivary fluid (α-amylase 75 U/mL) was added to the raspberry extracts (10 g) and incubated at 37 °C with constant agitation at 170 rpm for 2 min. After 2 min of continuous stirring, 20 mL of simulated gastric fluid (SGF) was added to the mixture, and the pH was adjusted to 2.5 and stirred at 170 rpm speed (Figure 1) and incubated for 2 h at 37 °C. A 10 mL sample was collected, and enzymatic reactions were stopped by placing the samples on ice before storing them at −80 °C until further analysis. Subsequently, the simulated intestinal fluid (SIF) was added, and the pH was adjusted to 7.5 and then incubated for 2 h at 37 °C. After incubation, the digesta was stored at −80 °C until further analysis. Figure 1 illustrates the simulated in vitro gastrointestinal digestion procedure of raspberry extracts. 

The percentage of recovery and bioaccessibility under simulated gastric and intestinal conditions was calculated using the following Formulas (4) and (5)

(4) $$Recovery\;\left(\%\right)=\frac{Bgc\;}{Bnd}\;\times \;100$$

where *B_gc_* is the content of the gastric digesta, while *B_nd_* is the content in the undigested beads.

(5) $$Bioaccessibility\;\left(\%\right)=\frac{Bsi\;}{Bnd}\;\times \;100$$

where *B_si_* is the content in the intestinal digesta, and *B_nd_* is the content in the undigested beads.

### 2.6. Quantification of Cyanidin Compounds

Individual anthocyanin components were quantified using the method of by injecting raspberry extract (10 µL) into the Shimadzu HPLC (Shimadzu Prominence-i-LC-2030C 3D, Kyoto, Japan) system. The chromatographic separation was achieved on a Shimadzu C18 column (250 × 4.6 mm, particle size 5 µm) using a gradient elution of 0.1% formic acid (solvent A) and 0.1% formic acid in acetonitrile (solvent B), applied as follows: 0% B at 0 min, 45% B at 25 min, 60% B at 45 min, 0% B at 46 min and 0% B at 49 min. The column was kept at 30 °C at a flow rate of 0.6 mL/min. Chromatograms were read at 520 nm. The identification and quantification of anthocyanins were achieved using pure external standards. The limit of detection (LOD) is 15 mg/L for cyanidin-3-O-sophoroside and 0.06 mg/L for cyanidin-3-O-glucoside 

Respectively. The limit of quantification (LOQ) for cyanidin-3-O-sophoroside and yanidin-3-O-glucoside is 0.02 mg/L and 0.02 mg/L respectively. 

### 2.7. Antioxidant Activities (FRAP & ABTS)

The FRAP (Ferric Reducing Antioxidant power) assay for microbeads was performed following the method of Thaipong et al. [20]. The stock solution consisted of 300 mM acetate buffer (3.1 g sodium acetate in 16 mL acetic acid, pH 3.6), 10 mM TPTZ in 40 mM HCl, and 20 mM FeCl_3_. The working solution was freshly prepared in a 10:1:1 ratio by mixing 25 mL acetate buffer, 2.5 mL TPTZ, and 2.5 mL FeCl_3_, then warmed to 37 °C. For the assay, 75 μL of microbead extract was mixed with 1425 μL of FRAP working solution and incubated in the dark for 30 min. Following incubation, 200 μL of the reaction mixture was used to measure absorbance at 593 nm using a spectrophotometer. The Trolox standard curve (0–100 μmol/mL) was represented by the equation Y = 0.0065x + 0.1029 (R^2^ = 0.99) and used to calculate the FRAP activity of the samples. Results were expressed as mM Trolox equivalent per gram of dry matter (mM TEAC/g DM).

The ABTS [2,2-azino-bis (3-ethylbenzothiazoline-6-sulfonic acid)] radical scavenging activity of microbead extracts was evaluated following the method described by Mhlanga et al. [21]. To prepare the ABTS stock solution (7 mM), 0.0406 g of ABTS was dissolved in 10 mL of distilled water, while the potassium persulfate stock solution (2.6 mM) was prepared by dissolving 0.0141 g of potassium persulfate in 20 mL of distilled water. Equal volumes of both solutions were mixed and incubated in the dark for 12–16 h to generate the ABTS•^+^ radical. To prepare the ABTS working solution, 5 mL of the ABTS radical solution was diluted with 145 mL of phosphate buffer. Microbead extracts underwent serial dilutions (20–100 μL), with the solvent volume adjusted based on the amount of extract used (80–0 μL). Each diluted extract was then mixed with 200 μL of ABTS working solution in a 96-well microplate and incubated for 7 min. Absorbance was measured at 750 nm using a spectrophotometer against a blank. Results were expressed as IC_50_ (mg/mL), representing the extract concentration needed to scavenge 50% of ABTS•^+^ radicals.

### 2.8. Statistical Analysis

The data was analysed using SPSS 26.0 (Statistical Package for Social Science IBM SPSS Statistics). The means were presented as ±SD (standard deviation). The analysis was carried out in triplicates throughout, and experiments were repeated twice. Data was analysed using one-way ANOVA and means were separated based on Turkey’s HSD (*p* ˂ 0.05).

## 3. Results

### 3.1. Encapsulation Efficiency, Retention of Anthocyanins in Microbeads

**Figure 2:**
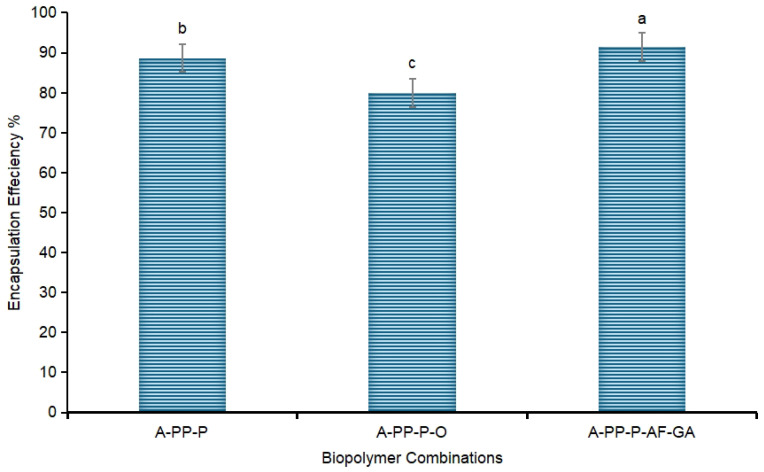
The anthocyanin encapsulation efficiency of microbeads. Letters above the vertical bars denote significant differences (*p* ˂ 0.05). Vertical bars indicate the standard deviation of treatment means. (a) A-PP-P: alginate/pea protein isolate/*psyllium mucilage*; (b) A-PP-P-O: alginate/pea protein isolate/*psyllium mucilage*/okra mucilage; (c) A-PP-P-AF-GA: alginate/pea protein isolate/*psyllium mucilage*/*Aloe ferox* gel/gallic acid.

**Figure 3:**
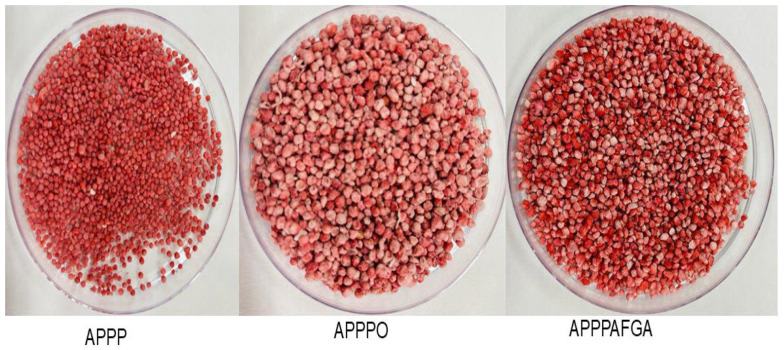
Encapsulated microbeads containing raspberry powder based on combinations of alginate/pea protein isolate/*psyllium mucilage*. Key: A-PP-P: alginate/pea protein isolate/*psyllium mucilage*; A-PP-P-O: alginate/pea protein isolate/*psyllium mucilage*/okra mucilage; A-PP-P-AF-GA: alginate/pea protein isolate/*psyllium mucilage* + *Aloe ferox* gel/gallic acid.

For all microbeads obtained (Figure 2), EE exceeded approximately 70%, but the highest EE (91.60%) was found for encapsulated (A-PP-P-AF-GA), microbeads, indicating their suitability as carriers for encapsulating raspberry powder anthocyanins. The control (A-PP-P at 88.45%) and the lowest EE% recorded for A-PP-P-O at 69.94%. The encapsulation materials have an impact on EE and the retention of anthocyanins. Different microbeads are shown in Figure 3. In addition, the interaction between polysaccharides and hydroxyls in raspberry extract facilitates hydrogen bonding and linkage with carboxylic acid groupings [8] and this could be strengthened by the inclusion of gallic acid into the biopolymer. The formation of hydrogen bonds between alginate and phenolic acids (Tannic acid) has been reported [22]. In addition, *psyllium mucilage* protein moiety enhanced the encapsulation efficiency of Natal plum polyphenols [8]. The hydrogen bonding between gallic acid and the binary polymer mixture would contribute to this molecular association

### 3.2. Scanning Electron Microscopy (SEM)

**Figure 4:**
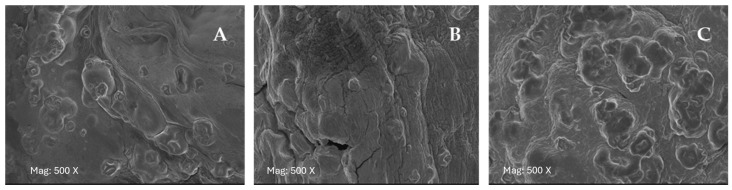
Scanning electron microscopy microstructure of raspberry microbeads. (**A**) A-PP-P: alginate/*psyllium mucilage*/pea protein isolate; (**B**) A-PP-P-O: alginate/pea protein isolate/*psyllium mucilage*/okra mucilage; (**C**) A-PP-P-AF-GA: alginate/pea protein isolate/*psyllium mucilage*/Aloe ferox gel/gallic acid.

Scanning electron microscopy (SEM) was used to determine the morphological structures of microbeads formulated with various biopolymer combinations (A-PP-P, A-PP-P-O, and A-PP-P-AF-GA) at 500× magnification. Figure 4A–C presents the morphologies of these various raspberry-rich microbeads. Typically, all encapsulated microbeads revealed uneven shrinkage, smooth-rough, homogeneous surfaces, and a spherical shape with minimal wall cracks or collapse [23]. Upon comparing the treatments (A-PP-P, A-PP-P-O, and A-PP-P-AF-GA) variations in particle structure can be seen visually on the images above (Figure 3). The morphological characteristics of microbeads are critical as they directly influence the solubility and release properties, consequently, their application potential in the food technology industry.

Alginate, pea protein isolate, and *psyllium mucilage* were used as primary ingredients in all treatments, which likely enhanced the hydrogel network strength throughout the freeze-drying process, preventing more shrinkage and collapse of the microbeads [24]. Encapsulation of raspberry extract with alginate demonstrated spherical-shaped microbeads with compact structures, likely due to the interaction between alginate and other biopolymers, including *psyllium mucilage,* okra mucilage, pea protein isolate, *Aloe ferox* gel, and gallic acid. These interactions resulted in homogenous morphological structures across all microbead treatments. Despite this, slight indentations and wrinkling were observed on the surface of all treatments (Figure 4A–C), which are consistent with findings in the literature [24]. The continuous and thick structure observed in all treatments may also be attributed to the synergistic interaction between alginate, pea protein isolate, and other biopolymers such as *psyllium mucilage*. Moreover, after adding gallic acid (Figure 4C) microbeads start to have a foggy background compared to those that don’t have gallic acid (A-PP-P and A-PP-P-O). It can be explained by interactions between polymer chains and gallic acid molecules incorporated into microbeads [25]. Additionally, the incorporation of Gallic acid in A-PP-P-AF-GA, comparatively enhanced surface morphology of the coating compared to A-PP-P and A-PP-P-O. These results indicate that the specific combination of biopolymers contributes to the structural integrity of the microbeads, making them suitable for application in food technology. The observations in this study align with Tang et al. [24] on the structural characterisation and stability of the pigment in microcapsules.

### 3.3. FTIR Spectra

**Figure 5:**
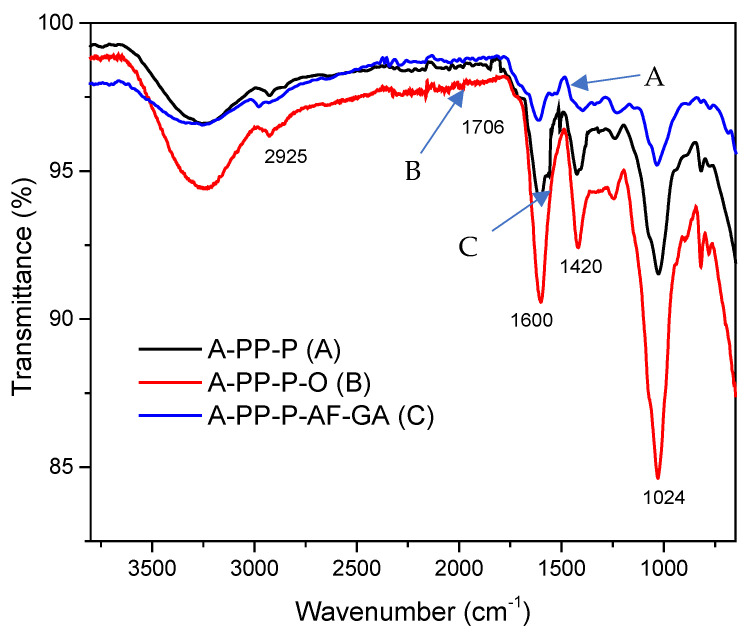
Fourier Transform Infrared Spectroscopy (FTIR) of raspberry microbeads. Key: A-PP-P: alginate/*psyllium mucilage*/pea protein isolate; A-PP-P-O: *psyllium mucilage*/okra mucilage/pea protein isolate/alginate; A-PP-P-AF-GA: *Aloe ferox* gel/*psyllium mucilage*/pea protein isolate/gallic acid/alginate.

FTIR spectra in Figure 5, reveal the chemical structures of raspberry-enriched alginate microbeads across different treatments (A–C). FTIR analysis was employed to identify the functional groups present in these microbeads. All microbeads showed a broad stretching vibration of the hydroxyl group (-OH) was observed within the range 3380–3000 cm^−1^ in all samples due to the presence of hydroxyl groups in the alginate polymeric structure [25]. The characteristic asymmetric and symmetric stretching vibrations of the carboxylate groups within the alginate backbone were observed at 1701 cm^−1^ (O-C=O) [25]. Peaks at 1624 cm^−1^ (C=C aromatic), confirmed the presence of phenolic compounds within the alginate structure [26]. Additionally, absorption at 1460 cm^−1^ was attributed to C–H stretching and aromatic ring deformation [26]. Peaks corresponding to the antisymmetric stretching of the carboxyl group were observed at 1420 cm^−1^, while the stretching vibrations of the C-O-C side groups appeared at 1024 cm^−1^ and 940 cm^−1^ [26]. Moreover, 2390 and 2918 cm^−1^ absorption bands were linked to the methyl group’s deformation and stretching within the mucilage C-H bond [27]. The peak at 1024 cm^−1^ was attributed to overlapping C-O and C-O-C stretching vibrations of glycosidic linkages in alginate [28]. Treatment A-PP-P-AF-GA had fewer peaks than other treatments (A-PP-P and A-PP-P-O), this could be due to several factors such as the chemical reaction during the breaking down of the bonds responsible for a particular vibrational mode, the strong hydrogen bond interactions between molecules within the matrix and molecules within the matrix having high symmetry. However, based on Figure 4, the addition of okra mucilage to the base, has not changed any interactions to the treatment as they had performed similarly. Overall, the FTIR spectra confirm that the functional groups in raspberry-enriched alginate microbeads are consistent with known structural characteristics of the components, validating the successful encapsulation of bioactive compounds within the alginate matrix. The characteristic spectra presented in this study are similar to those observed by Soiklom et al. [29] who observed spectral bands of anthocyanin at 3450–3100 cm^−1^, 2925 cm^−1^, 1706 cm^−1^, 1688–1600 cm^−1^ on the anthocyanin-rich gel beads from the extract of black rice which confirmed the effective encapsulation of anthocyanin in all the matrices. The slight change in the broadness or intensity of the hydroxyl stretching vibration (3380–3000 cm^−1^) is also noted which could indicate a strong interaction, such as hydrogen bonding in this case. However, no significant change in the C-O-C stretching around 1024 cm^−1^ is observed in the spectrum of A-PP-P–AF-GA corresponds to the glycosidic linkages in alginate. The FTIR spectrum of A-PP-P-O showed that after encapsulation, the treatment, original structures were not altered. However, the addition of *Aloe ferox gel* and gallic acid caused other bonds to disappear. Notable changes in the FTIR spectra of different encapsulation matrices were observed, including the disappearance, displacement, and intensity shifts of certain peaks, possibly due to interactions between the raspberry bioactive compounds and the alginate matrix [25].

### 3.4. X-Ray Diffraction

**Figure 6:**
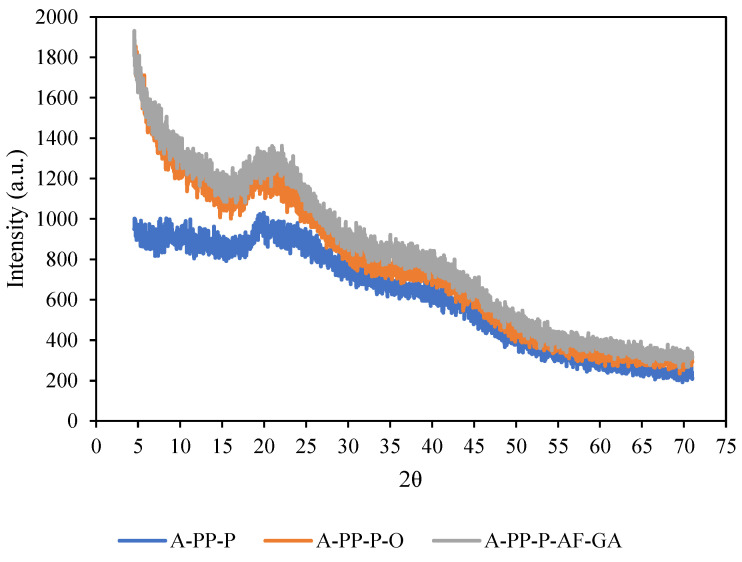
Xray Diffraction (XRD) of raspberry microbeads. Key: -PP-P: alginate/*psyllium mucilage*/pea protein isolate; A-PP-P-O: alginate/pea protein isolate/*psyllium mucilage*/okra mucilage; A-PP-P-AF-GA: alginate/pea protein isolate/*psyllium mucilage*/*Aloe ferox* gel/gallic acid.

X-ray diffraction (XRD) analysis was conducted to examine the effect of encapsulation of raspberry extract within alginate microbeads on their crystallinity. Figure 6 presents the XRD patterns for various treatments, including A-PP-P (control), A-PP-P-O, and A-PP-P-AF-GA. The XRD patterns reveal that treatments A-PP-P-O and A-PP-P-AF-GA maintained similar diffractograms, showing one major broad peak centred around 2 θ angle of 21.6° which is in agreement with the comparable amorphous structure reported for alginate [30]. In contrast, control A-PP-P showed lower intensity peaks, indicating more amorphous structure than the other treatments. The absence of sharp peaks in the diffractograms of different encapsulated beads indicates that the encapsulated anthocyanins are in an amorphous matrix, consistent with findings by Seke et al. [8]. The crystallinity index (CI) for each formulation was calculated by measuring the intensity and width of the crystalline and amorphous peaks in the XRD patterns where a higher CI value indicates a more crystalline structure. Among the treatments, A-PP-P-AF-GA exhibited the highest crystallinity (CI = 42.3%), with a more defined crystalline peak, indicating a more ordered molecular structure compared to the other treatments A-PP-P-O (CI = −39.1%) and A-PP-P (CI = 35%). The higher crystallinity observed in A-PP-P-O and A-PP-AF-GA could be attributed to structural changes occurring during the gelation process, potentially influencing the molecular arrangement within the microbeads [31]. Furthermore, variations in intermolecular interactions between the polymer matrix and the raspberry anthocyanins could contribute to the differences in crystallinity [16].

Treatment A-PP-AF-GA exhibited relatively higher crystallinity than other treatments, supporting observations made in the SEM analysis, which further strengthens the correlation between SEM and XRD results. These findings are consistent with the results reported by Ambrosi et al. [32], who studied the encapsulation of apple polyphenols in β-CD nanosponges, demonstrating amorphous characteristics in the encapsulation process. This study highlights the impact of polymer interactions and gelation conditions on the structural properties of anthocyanin-loaded microbeads.

### 3.5. In Vitro Digestion, Total Anthocyanins and Cyanidin Compounds

The changes in cyanidin compounds in raspberry-rich microbeads during in vitro digestion are shown in Table 2. Normally, anthocyanins in plants are glycosylated, and cyanidin-3-O-sophoroside, and cyanidin-3-O-glucoside are major compounds in red raspberry juice powder [11]. The TAC ranged from 1051.73 to 1429.88 mg *cyanidin-3-glucoside* equivalents (C3G) per 100 g^−1^ in undigested samples. Undigested samples microencapsulated with A-PP-P-AF-GA showed the highest TAC at 1429.88 mg C3G 100 g^−1^, followed by A-PP-P-O (1352.46 mg C3G 100 g^−1^), A-PP-P (1251.73 mg C3G 100 g^−1^) and the R-B-J-P (1150.2 mg C3G 100 g^−1^). During the gastric phase, TAC was recovered in the following order from the microbeads produced using different encapsulation matrices: A-PP-P-AF-GA > A-PP-P-O > A-PP-P > R-B-J-P. In the intestinal phase, A-PP-P-AF-GA microbeads released the most TAC (777.15 mg C3G 100 g^−1^), followed by A-PP-P-O microbeads, while R-B-J-P and A-PP-P released the least amount.

The major cyanidin compounds in raspberry juice extract were cyanidin-3-O-sophoroside and cyanidin-3-O-glucoside. In the gastric phase, A-PP-P-AF-GA microbeads showed the highest recovery of cyanidin-3-O-sophoroside (81.16%) and cyanidin-3-O-glucoside (79.3%) compared to other biopolymer combinations and R-B-J-P. Comparing A-PP-P-AF-GA with the other treatments and R-B-J-P, in the intestinal phase, A-PP-P-AF-GA had the highest bioaccessibility of cyanidin-3-O-sophoroside (54.61%) and cyanidin-3-O-glucoside (55.30%) (Table 3).

These results suggest that the specific combination of *Aloe ferox* gel and gallic acid within the alginate-based matrix significantly enhanced anthocyanin protection and controlled release under simulated digestive conditions. This enhanced performance aligns with FTIR findings, which showed notable changes in the hydroxyl stretching region and the disappearance or shift of certain peaks. Such spectral alterations indicate strong interactions—likely hydrogen bonding—between the alginate matrix and bioactive compounds. The preservation of glycosidic C–O–C linkages suggest the core alginate structure remained intact, while the modified surface chemistry due to *Aloe ferox* and gallic acid likely contributed to improved stability and bioaccessibility of the anthocyanins during digestion.

The results also indicate a higher recovery of cyanidin-3-O-sophoroside when compared to cyanidin-3-O-glucoside in general which could depend on the structural factors such as glycosidic linkage, molecular weight, and solubility. Cyanidin-3-O-glucoside has a glucose molecule whereas cyanidin-3-O-sophoroside, on the other hand, has a sophorose (a disaccharide composed of glucose and fructose) linked to cyanidin at the 3-position [33]. Sophorose glycosidic linkage might be more resistant to acidic and enzymatic hydrolysis due to its higher steric hindrance, which could explain its higher recovery and stability [33,34]. The larger molecular size of cyanidin-3-O-sophoroside, may also confer greater stability and lower solubility and reduced susceptibility to enzymatic degradation and could explain the better recovery rate and bioaccessibility observed for cyanidin-3-O-sophoroside [35].

As a result of the wall material reducing phytochemical loss in gastric transit, 58.48% of the anthocyanins in strawberry juice powder (encapsulated pea protein isolate + okra mucilage + *psyllium mucilage*) were bioaccessible [8]. The low pH of the gastric medium increases the stability of total anthocyanins and individual anthocyanins due to the formation of flavylium cations [1]. Anthocyanin indigestibility is related to alkaline pH, which increases non-flavylium cation forms, as well as anthocyanin binding to proteins and bile salts, which results in indigestible complexes [31] and carbinol, a pseudobasic substance [36]. There is a lower concentration of anthocyanins in the intestinal phase due to the degradation of cyanidin derivatives into protocatechuic acid and phloroglucinol [37]. It is assumed that anthocyanin consumption is around 12.5 mg day^−1^ in the US. It ranges from 19 to 65 mg day^−1^ for men and 18–44 mg day^−1^ for women in Europe. Australia reports an average intake of 24 mg day^−1^, while Finland reports up to 150 mg day^−1^ [38]. Due to anthocyanins proven health properties, consuming more fresh fruits and vegetables will boost antioxidant levels and protect against chronic and degenerative diseases [38].

Polyelectrolyte Complexes can be formed through electrostatic interactions between oppositely charged polyelectrolytes, such as Aloe ferox extract (containing various compounds) and gallic acid. Gallic acid interacts with anthocyanins, increasing their colour stability and preventing degradation. The polyelectrolyte complex acts as a carrier, encapsulating the anthocyanins and further protecting them from environmental factors like pH changes during in vitro digestion. The results indicate that the presence of *Aloe ferox* and gallic acid components in A-PP-P-AF-GA protected these anthocyanins from degradation based on the recovery rate percentage in the gastric phase. Gallic acid is a known antioxidant with a strong ability to scavenge free radicals and prevent oxidative stress [39]. Similarly, protective effect of *Aloe ferox* against oxidatively stress, significantly reducing the levels of lipid peroxidation and reactive oxygen species (ROS) was previously reported [40].

Comparatively, our results are higher (60.91%)/lower (67.06%) than those presented by Seke et al. [8]. Thus, the findings of this study provide important insights into optimizing anthocyanin delivery through biopolymer-based encapsulation systems for functional food development. Jang & Koh [7] reported that maltodextrin (MD), or MD plus carboxymethyl cellulose (CMC), gum Arabic (GA), or xanthan gum (XG) encapsulation alone or in combination increased cyanidin-3-O-glucoside concentration in the intestinal phase by 38 to 40 mg 100 g^−1^ which is lower than all different biopolymer combinations included in this study.

### 3.6. Antioxidant Activity of Raspberry-Rich Microbeads

**Figure 7:**
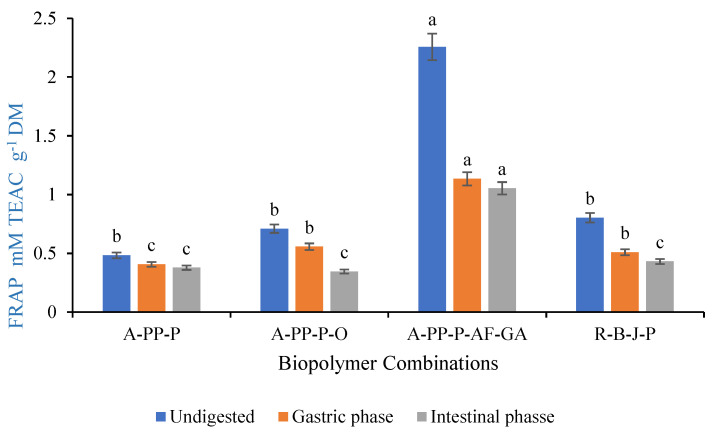
Ferric reducing antioxidant power of freeze-dried microbeads. Letters above the vertical bars denote significant differences (*p* ˂ 0.05). Letters above the vertical bars denote significant differences (*p* ˂ 0.05). Vertical bars indicate the standard deviation of treatment means. Key: A-PP-P-alginate/pea protein isolate/*psyllium mucilage*; A-PP-P-O- alginate/pea protein isolate/*psyllium mucilage*/okra; A-PP-P-AF-GA- alginate/pea protein isolate/*psyllium mucilage*/*Aloe ferox* gel/gallic Acid, R-B-J-P- raspberry juice powder.

The FRAP values in undigested samples ranged between 0.48- and 2.26-mM TEAC g^−1^, with A-PP-P-AF-GA demonstrating the highest FRAP activity (2.26 mM TEAC g^−1^), followed by R-B-J-P (0.80 mM TEAC g^−1^), A-PP-P-O (0.71 mM TEAC g^−1^), and the lowest was A-PP-P (0.48 mM TEAC g^−1^) (Figure 7). Adding *Aloe ferox* and gallic acid to A-PP-P increases antioxidant power in undigested samples, at the gastric and intestinal phases, when compared with A-PP-P, A-PP-P-O, and non-encapsulated R-B-J-P. Furthermore, A-PP-P-AF-GA maintained the highest FRAP during the intestinal phase, at 1.05 mM TEAC g^−1^, compared to all other treatments. Gallic acid showed the highest FRAP value among hydroxybenzoic phenolic acids [36]. There are three OH groups located at positions 3, 4, and 5 in the chemical structure of gallic acid that may explain its high reducing power [41]. All treatments reduced FRAP activity during digestion, due to polyphenols’ sensitivity to pH changes and digestive enzymes, which impair their stability and antioxidant properties [42]. This indicates that A-PP-P-AF-GA microencapsulation protects anthocyanins from pH changes, making them suitable for post-harvest food applications. The freeze-drying process further stabilizes these bioactive compounds, making them suitable for long-term storage and integration into food products. Our finding suggests that raspberry juice powders coated with A-PP-P-AF-GA (microbeads) could play an essential role in promoting human health benefits.

**Figure 8:**
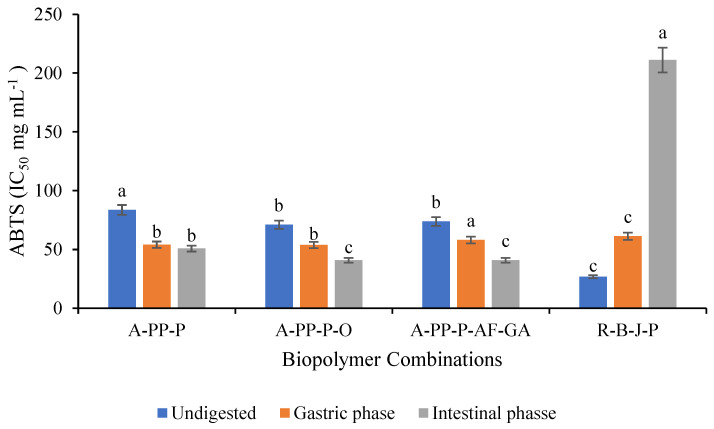
ABTS scavenging activity of freeze-dried microbeads. Letters above the vertical bars denote significant differences (*p* ˂ 0.05). Vertical bars indicate the standard deviation of treatment means. Key: A-PP-P-alginate/pea protein isolate/*psyllium mucilage*; A-PP-P-O- alginate/pea protein isolate/*psyllium mucilage*/okra; A-PP-P-AF-GA- alginate/pea protein isolate/*psyllium mucilage*/*Aloe ferox* gel/gallic Acid, R-B-J-P- raspberry juice powder.

The ABTS scavenging activity of undigested and unencapsulated R-B-J-P (IC_50_ 26.79 mg mL^−1^) powder was greater than that of encapsulated microbeads A-PP-P-AF-GA (IC_50_ 73 mg mL^−1^), A-PP-P-O (IC_50_ 71 mg mL^−1^), and A-PP-P (IC_50_ 83.65 mg mL^−1^) in undigested samples. (Figure 8). This could be due to the deficient extraction of phenolic compounds in microbeads and the extract’s reaction with the capsule membrane, leading to reduced antioxidant activity [43]. In contrast, the unencapsulated R-B-J-P powder showed the highest IC_50_ (211.13 g mL^−1^) indicating the lowest antioxidant activity in the intestinal phase while both encapsulated micro beads showed comparatively higher ABTS scavenging activity. Amongst the encapsulated samples A-PP-P-AF-GA (IC_50_ 40.79 mg mL^−1^ and A-PP-P-O (IC_50_ 40.74 mg mL^−1^) exhibited the higher antioxidant activity than A-PP-P in the intestinal phase. In contrast to unencapsulated extracts, microbeads delay the release of bioactive compounds, protecting them from degradation. Encapsulation can increase stability, protect bioactive compounds from harsh conditions such as pH, and control the release of bioactive compounds. Variations in antioxidant activity are influenced by the type of polyphenols present, encapsulation materials used, and physiological conditions encountered during digestion [44].

## 4. Conclusions

The study successfully developed raspberry-rich anthocyanin microbeads using various biopolymer combinations including alginate, *psyllium mucilage*, *Aloe ferox* gel, okra mucilage, pea protein isolate, and gallic acid, at different concentrations, assessing their stability and chemical properties before and after digestion. Biopolymer matrix with alginate, pea protein isolate, *psyllium mucilage, Aloe ferox* gel and gallic acid performed better than other matrices andwere the most suitable for further application. This formulation showed the highest crystallinity index, superior encapsulation efficiency, and enhanced antioxidant stability after digestion, making it the optimal candidate for use in food systems. The anthocyanin-rich compound from raspberry fruit extract showed a high encapsulation efficiency percentage. These microbeads, rich in antioxidant properties, have potential in the food industry as natural colorants. Anthocyanins are associated with several health-promoting effects, including anti-inflammatory, anti-cancer, and cardiovascular-protective properties. Microencapsulation protects anthocyanins from environmental factors, making them suitable for post-harvest food applications. The freeze-drying process further stabilizes these bioactive compounds, making them suitable for long-term storage and integration into food products such as beverages, dairy, and bakery items. This suggests that raspberry microbeads could play an essential role in improving food quality, extending shelf life, and promoting human health.

## Figures and Tables

**Table 1:** Treatment formulations.

Treatments	Samples	Code
**Treatment 1**	2% Alginate + 1.5% Pea protein + 1% *Psyllium mucilage* (Control)	(A-PP-P)
**Treatment 2**	2% Alginate + 1.5% Pea protein + 1% *Psyllium mucilage*+ 1% Okra	(A-PP-P-O)
**Treatment 3**	2% Alginate + 1.5% Pea protein + 1% *Psyllium mucilage*+ 2% *Aloe ferox gel* + 1% Gallic acid	(A-PP-P-AF-GA)

**Table 2:** The effect of invitro digestion on the total anthocyanin content of raspberry-rich microbeads.

Total Anthocyanin Concentration (mg C3G 100 g ^−1^) dw				
Encapsulation Matrices to Produce Microbeads of Raspberry Juice Powder	Undigested	Gastric Phase	Recovery %	Intestinal Phase
A-PP-P	1251.73 ^c^ ± 0.97	630.25 ^c^ ± 0.32	50.35 ^bc^ ± 0.90	427.48 ^c^ ± 0.89
A-PP-P-O	1352.46 ^b^ ± 0.61	742.00 ^b^ ± 0.02	54.86 ^b^ ± 0.52	609.19 ^b^ ± 0.62
A-PP-P-AF-GA	1429.88 ^a^ ± 0.51	870.94 ^a^ ± 0.57	60.91 ^a^ ± 0.42	777.15 ^a^ ± 0.17
R-B-J-P	1150.2 ^d^ ± 0.35	542.43 ^d^ ± 0.15	47.16 ^c^ ± 0.91	435.86 c ± 0.23

Means values were calculated based on Turkey’s HSD test in triplicate denoting the significant difference within the same column. Means followed by the same letter within the bio-accessibility column is not significantly different at *p* < 0.05. ±SD standard deviation. Key: A-PP-P- alginate/pea protein isolate/*psyllium mucilage*; A-PP-P-O- alginate/pea protein isolate/*psyllium mucilage*/okra; A-PP-P-AF-GA- alginate/pea protein isolate/*psyllium mucilage*/*Aloe ferox* gel/gallic Acid. R-B-J-P- Raspberry juice powder.

**Table 3:** The changes in cyanidins in raspberry-rich microbeads during in vitro digestion.

Biopolymers	Undigested Raspberry Microbeads	Gastric Phase	Recovery %	Intestinal Phase	Bio-Accessibility %
Cyanidin-3-O-sophoroside (mg 100 g^−1^)					
A-PP-P	930.94 ^a^ ± 9.04	679.28 ^b^ ± 4.79	72.96	417.49 ^c^ ± 7.43	44.85 ^b^ ± 1.02
A-PP-P-O	962.46 ^a^ ± 5.28	709.04 ^b^ ± 7.93	73.67	433.11 ^c^ ± 3.06	45.00 ^b^ ± 0.52
A-PP-P-AF-GA	994.02 ^a^ ± 8.23	806.79 ^b^ ± 3.44	81.16	542.88 ^c^ ± 8.16	54.61 ^a^ ± 0.83
R-B-J-P	864.99 ^a^ ± 0.27	295.59 ^d^ ± 0.50	34.17	266.43 ^d^ ± 0.95	30.80 ^c^ ± 0.31
Cyanidin-3-O-glucoside (mg 100 g^−1^)					
A-PP-P	68.62 ^a^ ± 2.71	39.93 ^c^ ± 0.59	58.19 ^bc^	21.67 ^d^ ± 0.52	31.58 ^c^ ± 0.32
A-PP-P-O	76.45 ^a^ ± 3.01	52.65 ^b^ ± 0.41	68.87 ^b^	37.18 ^c^ ± 0.21	48.63 ^b^ ± 0.27
A-PP-P-AF-GA	115.23 ^a^ ± 2.13	91.39 ^a^ ± 0.89	79.3 ^a^	63.72 ^b^ ± 0.81	55.30 ^a^ ± 0.17
R-B-J-P	97.16 ^a^ ± 0.10	32.16 ^c^ ± 0.27	33.10 ^d^	29.77 ^d^ ± 0.77	30.64 ^c^ ± 0.15

Means values were calculated based on Turkey’s HSD test in triplicates denoting the significant difference within the same column. Means followed by the same letter within the row is not significantly different at *p* < 0.05. ±SD-Standard deviation: A-PP-P-alginate/pea protein isolate/*psyllium mucilage*; A-PP-P-O- alginate/pea protein isolate/*psyllium mucilage*/okra; A-PP-P-AF-GA- alginate/pea protein isolate/*psyllium mucilage*/*Aloe ferox* gel/gallic acid, R-B-J-P- Raspberry juice powder.

## Data Availability

The datasets generated for this study are available on request to the corresponding author.
